# Safety Assessment of Concurrent Vaccination with the HPV Vaccine and the COVID-19 Vaccine in Fujian Province, China: A Retrospective Study

**DOI:** 10.3390/vaccines12060673

**Published:** 2024-06-18

**Authors:** Yan Zhang, Yuhang Zhang, Binhua Dong, Wenyu Lin, Yuxuan Huang, Kelvin Stefan Osafo, Xite Lin, Tingting Jiang, Yu Zhang, Huachun Zou, Pengming Sun

**Affiliations:** 1 The State Key Laboratory of Molecular Vaccinology and Molecular Diagnostics, National Institute of Diagnostics and Vaccine Development in Infectious Diseases, School of Public Health, Xiamen University, Xiamen 361102, China; yaaynn1030@stu.xmu.edu.cn (Y.Z.); zyhxmu20240117@stu.xmu.edu.cn (Y.Z.); 2 Laboratory of Gynecologic Oncology, Fujian Maternity and Child Health Hospital, College of Clinical Medicine for Obstetrics & Gynecology and Pediatrics, Fujian Medical University, Fuzhou 350001, China; dongbinhua86@fjmu.edu.cn (B.D.); lwy@fjmu.edu.cn (W.L.); huangyuxuan@fjmu.edu.cn (Y.H.); osafokelvinstefan@outlook.com (K.S.O.); linxite@fjmu.edu.cn (X.L.); jtt1998@fjmu.edu.cn (T.J.); 3 Fujian Clinical Research Center for Gynecologic Oncology, Fujian Maternity and Child Health Hospital, Fuzhou 350001, China; 4 Fujian Key Laboratory of Women and Children’s Critical Diseases Research, Fujian Maternity and Child Health Hospital, Fuzhou 350001, China; 5 Department of Gynecology, Xiangya Hospital, Central South University, Changsha 410008, China; xyzhangyu@csu.edu.cn; 6 Gynecological Oncology Research and Engineering Center of Hunan Province, Changsha 410008, China; 7 School of Public Health, Fudan University, Shanghai 200433, China; 8 Fujian Maternity and Child Health Hospital, College of Clinical Medicine for Obstetrics and Gynecology and Pediatrics, Fujian Medical University, Fuzhou 350001, China; 9 School of Public Health, Xiamen University, Xiamen 361102, China

**Keywords:** human papillomavirus, HPV vaccine, COVID-19 vaccine, concurrent, immunizations, safety

## Abstract

During acute respiratory infections, women may concurrently receive human papillomavirus (HPV) and respiratory vaccines, as observed during the coronavirus disease 2019 (COVID-19) pandemic in China. However, few studies have assessed the safety of such concurrent administration, which could impact HPV vaccination schedules. This study analyzes the safety and optimal sequence of concurrent HPV and COVID-19 vaccinations. For this purpose, we surveyed women with both vaccines from January to October 2023 in Fujian Province, China. During this process, we collected vaccination history and adverse event (AE) data via telephone or interviews. Participants were grouped as Before, Concurrent, or After based on their vaccination sequence. A Chi-squared test, exact Fisher tests, and logistic regression were used to analyze the incidence of AEs and factors influencing vaccine safety. Overall, 1416 eligible participants were included. Although overall AE risk with the HPV vaccine was unaffected by vaccination sequence, individual AEs varied statistically between groups, including pain at the vaccination site (*p* < 0.001) and prolonged menstruation duration (*p* = 0.003). Based on the results, the optimal sequence would be to receive the HPV vaccine after the COVID-19 vaccine (After group). This insight may guide future emergency vaccination sequences for HPV and other respiratory infectious diseases.

## 1. Introduction

Since 2022, five human papillomavirus (HPV) vaccines have been licensed by the Chinese Food and Drug Administration (CFDA) and used in mainland China, while four HPV vaccines have been licensed for use in other countries [1,2]. The efficacy of HPV vaccines in preventing cervical cancer and precancerous lesions is well recognized [3]. Previous studies have shown that, among young women, the risk of cervical cancer after receiving the HPV vaccine is 88% lower than that before vaccination [4,5,6]. Generally speaking, it takes six months to receive the HPV vaccine fully. During these six months, women may receive other vaccines concurrently [7,8]. In particular, during an outbreak of an acute infectious disease, women receiving the HPV vaccine may need to receive a vaccine for the infectious disease concurrently.

Since the coronavirus disease 2019 (COVID-19) outbreak in 2019, respiratory diseases have had the highest incidence rate by transmission route (340.95 cases per 100,000 people) [9]. Consequently, there is increasing interest in respiratory infections as an issue of public health [10]. To quickly reduce the impact of the COVID-19 pandemic on public health, many countries developed COVID-19 vaccines as the primary tool to combat the disease. Since March 2021, China has regarded the COVID-19 vaccine as a key prevention strategy and organized universal vaccination. By April 2023, 90.6% of the population in China had been fully vaccinated against COVID-19, according to a report by the Chinese Center for Disease Control and Prevention. This program made the COVID-19 vaccine the respiratory infectious disease vaccine with the highest coverage in China, increasing demand for women to receive the HPV and COVID-19 vaccines concurrently.

However, there remains limited research on the safety of women who receive the HPV and COVID-19 vaccines concurrently. At the same time, previous studies have shown that women may experience adverse events (AEs), such as pain at the vaccination site and changes in their menstrual cycles after receiving the HPV vaccine. This factor also raises concerns about the safety of the vaccine. In addition, due to the outbreak of COVID-19 and the impact of long COVID-19, problems such as restricted travel and insufficient medical public resources remain a concern. Although COVID-19 has become a common respiratory disease, long COVID-19 may still seriously affect public health [11]. Finally, the HPV vaccine faces supply shortages in China. These problems indicate that, during periods of acute infectious diseases, women are likely to miss the opportunity to receive the HPV vaccine. Thus, HPV vaccination programs have become severely disrupted, which will increase the incidence of and have a severe negative impact on the prevention and control of cervical cancer in the future [12,13].

Most previous safety studies on vaccines were clinical trials [14,15,16] that involved some symptoms of short-term events, such as injection site pain, fever, and headache [14,15]. However, there remains a lack of data on long-term events such as menstrual irregularities. Therefore, conducting large-scale safety monitoring of the vaccine after its launch is crucial [17]. This study explores the safety of concurrent vaccination with the HPV and COVID-19 vaccines in the context of public health and explores the potential optimal vaccination sequence. As a method to combat a respiratory infectious disease, the COVID-19 vaccine may provide a reference for concurrent vaccination with HPV vaccines in the future.

## 2. Materials and Methods

### 2.1. Study Design

Fujian Province is one of the 34 provinces in China (23°31′–28°18′ N, 115°50′–120°43′ E). This province consists of 9 prefecture-level cities and is located in the coastal area of China with convenient transportation. In 2021, the total population of Fujian Province was approximately 42 million. Census data for the total population of Fujian were obtained from the seventh census. Compared with other provinces, Fujian Province has a higher HPV infection rate (33.3% vs. 16.4%) [18,19]. Moreover, the HPV vaccine was not included in the national immunization program in China. In China, HPV vaccination is a voluntary action that is chosen by individuals. On 12 May 2022, Fujian Province launched a free HPV vaccine program for women. This project provides free Bivalent HPV vaccines to girls aged 13–14 under the principles of being voluntary and free. Fujian Province contains the first cities that offer free HPV vaccines to girls of appropriate age with support from the provincial government.

From January to October 2023, we selected two vaccination sites (Fuzhou Adult Immunization Clinic and Jinan District Maternal and Child Health Hospital) in Fujian Province, China for a retrospective descriptive study. Participants were surveyed verbally and interviewed. Participants were asked about their vaccination history and AEs with the HPV and COVID-19 vaccines. Participants were divided into three groups according to the sequence of vaccination: (1) Before group, (2) Concurrent group, and (3) After group. Comparing the incidence of AEs among the three groups of people, we analyzed the impact of different vaccination sequences on the safety of the two vaccines.

This study was approved by the Ethics Committee of Fujian Maternal and Child Health Hospital (2023KYLLR01025). This retrospective study is registered with ClinicalTrials.gov, NCT05932576, and followed the Strengthening the Reporting of Observational Studies in Epidemiology (STROBE) reporting guidelines [20]. In this design section, the requirements of survey research guidelines such as the disposal of telephone follow-up results and the calculation of response rate were considered by us [21,22]. Verbal consent was obtained via telephone or interview for adults 18 years and older. For minors aged 9–18, informed consent was obtained from their legal guardians. Their legal guardians also reported the results of the investigation.

### 2.2. Criteria for Participants

Participants in this study came from two vaccination sites in the Fujian Province Vaccination Integrated Service Management Information System (FPVISMIS) (N = 9240). This study used a simple random sampling procedure. Through the SPSS 26.0 random number generator, researchers randomly selected 300 participants for pre-investigation. We pre-investigated the incidence of AEs of HPV, which was 26.2% in the Before group, 39.5% in the Concurrent group, and 14.6% in the After group—the sample ratio of the Before, Concurrent, and After groups was 2:1:1, 1 − β = 0.9, α = 0.05. The sample size was calculated using PASS 17.0, which is 400:200:173. Three attempts were made to contact each participant. Response rate 1 is the number of complete interviews divided by the number of interviews plus the number of non-interviews plus all cases of unknown eligibility [21,22]. Based on an 80% response rate 1, we estimated the sample size needed to be reached in the formal study (480 participants in the Before group, 240 participants in the Concurrent group, and 208 participants in the After group). After excluding the pre-investigation participants and considering our labor cost, we finally used a random number generator to extract 30% from the population for a formal study (n = 2682). After pre-testing the survey process, we discussed with obstetrics, gynecology, and public health experts to classify the symptoms using professional terms to help standardize the research. For example, we changed “pain in the arm” to “pain at the vaccination site”. We primarily asked about the occurrence of specific AEs. If the participant answered yes, we asked about the duration and severity of the symptoms. The survey instrument and the descriptive baseline information of the study participants (n = 2682) are can be found in the Supplementary Materials (Tables S1 and S2).

All participants in this study were from Fujian Province, China. Before the survey began, all participants were informed of the purpose and procedures of this study, and informed consent was obtained. Participants met the following criteria: (1) Women aged 9–45; (2) having voluntarily completed all immunization procedures for the HPV vaccine and the COVID-19 vaccine before the survey began with their vaccine-related information (for example, the participant identity card [ID], name, date of birth (age), residence, vaccine name, vaccination manufacturer, vaccination date and previous history of HPV infection) recorded by FPVISMIS; and (3) willing and able to cooperate with investigations and independently answer relevant questions.

### 2.3. Definition of Variables

The first large-scale vaccination against COVID-19 in China began in March 2021. Women who received the first dose of the COVID-19 vaccine more than one month after completing the whole HPV vaccination procedure were defined as receiving the HPV vaccination before COVID-19 vaccination (Before group). Women who received any dose of the COVID-19 vaccine during the three-dose HPV vaccination program or received any dose of the HPV vaccine between March 2021 and January 2022 were defined as receiving concurrent vaccination with the HPV vaccine and the COVID-19 vaccine (Concurrent group). Women who received the first dose of the HPV vaccine more than one month after completing the COVID-19 vaccination procedure were defined as receiving the HPV vaccination after the COVID-19 vaccination (After group).

Using a telephone survey, two trained researchers surveyed participants on all AEs of receiving the HPV and COVID-19 vaccines. The HPV vaccines included Cecolin^®^, Cervarix^®^, Gardasil^®^, and Gardasil 9^®^, while the COVID-19 vaccines included two inactivated vaccines (CoronaVac^®^ and COVILO^®^). AEs referred to all adverse medical events after participants received a vaccine, including symptoms, signs, or diseases that were not necessarily related to the vaccine [23]. Table S3 provides details on the definitions of AEs.

We evaluated local and systemic AEs after vaccination according to the Guidelines for Grading Standards of Adverse Events in Clinical Studies of Prophylactic Vaccines issued by the National Medical Products Administration (NMPA, located in Beijing, China) in 2019. According to the guidelines updated by the International Federation of Gynecology and Obstetrics (FIGO, located in London, UK) in 2018 [24], menstrual irregularities were specifically divided into eight symptoms: short cycle, long cycle, irregular menstrual cycles, prolonged menstrual duration, light menstrual bleeding, heavy menstrual bleeding (HMB), dysmenorrhea, and intermenstrual bleeding (IMB). If menstrual irregularities were reported, the researchers conducted a second follow-up with the affected participants after six months.

### 2.4. Statistical Analysis

Since Gardasil^®^ and Gardasil 9^®^ are manufactured by Merck Sharp & Dohme (located in Rahway, NJ, USA), they use the same adjuvant and medium for vaccine production. Consequently, these two vaccines were combined under the Gardasil^®^/Gardasil 9^®^ group for analysis. We used univariate analysis with Chi-squared or exact Fisher tests to explore potential factors affecting immunization and the incidence of AEs from vaccines received in the three sequences. Multivariable logistic regression using the forward likelihood ratio method was used to analyze factors associated with the occurrence of AEs. The data were analyzed statistically using IBM SPSS Statistics for Windows, version 26.0 (IBM Corp, Armonk, NY, USA). All *p* values were two-sided, α = 0.05. The Bonferroni method was used for pairwise comparison to analyze differences among the three groups.

## 3. Results

### 3.1. Flow Chart and Demographic Characteristics

**Figure 1:**
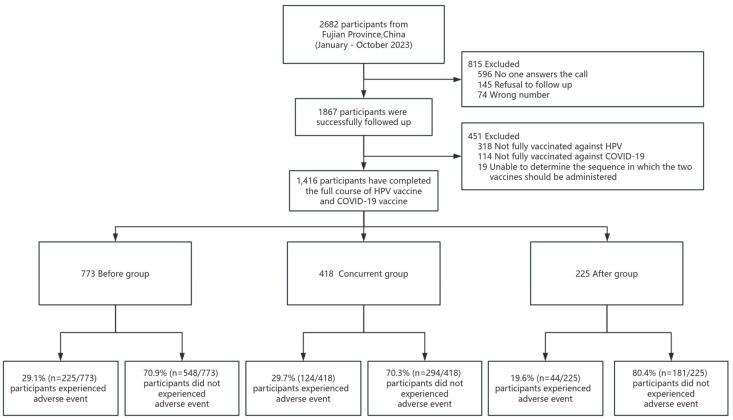
Flow chart of this study.

This study was conducted from January to October 2023 and excluded 145 women who refused to be surveyed, 74 women who had incorrect phone numbers, and 596 women who did not respond to the three telephone surveys. A total of 1867 women were successfully reached for follow-up. Response rate 1 was 69.6% (1867/2682). During the data analysis, we excluded 318 women who had not been fully vaccinated with the HPV vaccine, 114 women who had not been fully immunized with the COVID-19 vaccine, and 19 women who could not determine the sequence in which the two vaccines were administered. Ultimately, 1416 total participants were included in the analysis. These remaining participants had all completed a full vaccination course with the HPV and COVID-19 vaccines (Figure 1).

The average age of the women was 33 years old. Approximately 47.7% (675/1416) of the women were 29–38 years old, and 53.8% (762/1416) were vaccinated with Gardasil^®^/Gardasil 9^®^. In addition, 15.0% (213/1416) of participants experienced AEs after receiving two vaccines, while 55.9% (792/1416) did not experience AEs after receiving two vaccines. A further 12.7% (180/1416) of participants experienced AEs after receiving the HPV vaccine but had no AEs after receiving the COVID-19 vaccine (Occurred only with HPV). Finally, 16.3% (231/1416) of participants experienced AEs after receiving the COVID-19 vaccine but experienced no AEs after receiving the HPV vaccine (Occurred only with COVID-19; Table 1).

### 3.2. Specific AEs Assessment in Women Receiving the HPV Vaccine and COVID-19 Vaccines

Among all matched women, the incidence of overall AEs with only HPV vaccine administration was significantly lower than that with only COVID-19 vaccine administration (12.7% vs. 16.3%, *p* = 0.014), especially in terms of systemic AEs (6.7% vs. 10.6%, *p* < 0.001). However, there was no difference in the incidence of local AEs after vaccination between the two vaccines (*p* = 0.337). The incidence of systemic AEs after immunization with the HPV vaccine was lower than that with the COVID-19 vaccine. It mainly included issues with the respiratory system {fever (0.1% vs. 1.2%, *p* < 0.001), pain at non-vaccination sites (primarily sore throat, 0.1% vs. 0.6%, *p* = 0.039)}, issues with the brain {insomnia (0.0% vs. 0.5%, *p* = 0.016), drowsiness (0.6% vs. 2.7%, *p* < 0.001), fatigue (0.3% vs. 3.8%, *p* < 0.001)}, and immune system complications {allergic reaction (0.1% vs. 0.7%, *p* = 0.039)}. Notably, the incidence of menstrual irregularities that occurred only with the HPV group was higher than that that occurred only with the COVID-19 group (7.4% vs. 3.0%, *p* < 0.001; Table 2).

To explore specific symptoms associated with menstrual irregularities, we conducted a secondary follow-up with women who experienced such events. The results found that among 1416 women, the incidence of menstrual irregularities in the Occurred only with HPV group was higher than that in the Occurred only with COVID-19 group and mainly manifested as a prolonged menstrual duration (0.6% vs. 0.1%, *p* = 0.039). However, there were no differences in the incidence of other related symptoms after receiving the two vaccines (short cycle, long cycle, irregular menstrual cycles, light menstrual bleeding, HMB, dysmenorrhea, and IMB; Table 2).

### 3.3. AEs Associated with HPV Vaccine Based on Different Vaccination Sequences

To evaluate the impacts of different vaccination sequences on the safety of the HPV vaccine, we divided the 1416 women into three groups: 773 women were vaccinated with the HPV vaccine before the COVID-19 vaccine (Before group), 418 women received the HPV vaccine and the COVID-19 vaccine concurrently (Concurrent group), and 225 women were vaccinated with the HPV vaccine after the COVID-19 vaccine (After group) (Figure 1).

The results showed that the overall incidence of AEs with the HPV vaccine was statistically significant under the three different vaccination sequences (*p* = 0.011), which was mainly reflected in local AEs (*p* < 0.001) (Table 3). However, only anaphylaxis differed among systemic AEs (*p* = 0.026). After further pairwise comparisons, we found that the After group was significantly lower than the Before group {overall AEs (19.6% vs. 29.1%, *p* = 0.005) for local AEs (8.0% vs. 22.3%, *p* < 0.001)} (Table S4). The After group was also significantly lower than the Concurrent group {overall AEs (19.6% vs. 29.7%, *p* = 0.006) for local AEs (8.0% vs. 19.4%, *p* < 0.001)}. The incidence of local AEs in the After group was lower than in the Before and Concurrent groups, mainly manifested as pain at the vaccination site (7.1% vs. 21.1%, *p* < 0.001; 7.1% vs. 18.9%, *p* < 0.001). Although there were differences among systemic AEs in the incidence of anaphylaxis after receiving HPV vaccines in the three sequences, these differences were not statistically significant after multiple comparisons.

Although there were no differences in the incidence of menstrual irregularities among the three sequences of HPV vaccines, pairwise comparison showed that the incidence of prolonged menstrual duration in the Before group was lower than that in the After group (0.0% vs. 1.8%, *p* = 0.003; Table 3 and Table S4).

### 3.4. AEs Associated with the COVID-19 Vaccine Based on Different Vaccination Sequences

We found that the overall AE rate of the COVID-19 vaccine was statistically significant under the three different vaccination sequences (*p* < 0.001). Moreover, after age stratification, we observed differences in the overall incidence of AEs among the three groups of women aged 19–45 (*p* = 0.002; *p* < 0.001; *p* < 0.001)) (Table 4). After comparison, among women aged 19–45 years, the overall incidence of AEs in the After group was found to be lower than that in the Before group {19–28 years old (10.6% vs. 34.8%, *p* < 0.001); 29–38 years old (11.3% vs. 43.9%, *p* < 0.001); 39–45 years old (6.9% vs. 33.1%, *p* < 0.001)} (Table S5). These results suggest that age may be an influencing factor in AEs after COVID-19 vaccination.

Without age stratification, we observed differences in local AEs among the three sequences (*p* < 0.001), which mainly manifested as pain and swelling at the vaccination site (*p* < 0.001; *p* = 0.041). After multiple comparisons, we found that the After group had the lowest incidence of AEs related to pain at the vaccination site (24.6% vs. 16.5% vs. 5.8%, *p* < 0.001). Regarding swelling at the vaccination site, the incidence of AEs in the After group remained lower than that in the Before group (0.4% vs. 3.6%, *p* = 0.011). This study also found that the After group had the lowest incidence of systemic AEs (*p* < 0.001). The differences in the incidence of systemic AEs among the three groups mainly manifested as menstrual irregularities events (*p* = 0.026; Table 4), while the other events had no statistical significance.

Specifically, the incidence of menstrual irregularities in the After group was lower than in the Before group (0.9% vs. 4.9%, *p* = 0.006). However, after investigating related symptoms, we found that the differences between the three groups were not statistically significant (Table 4).

### 3.5. Multifactorial Regression Analysis of the Vaccine Influencing Factors

**Figure 2:**
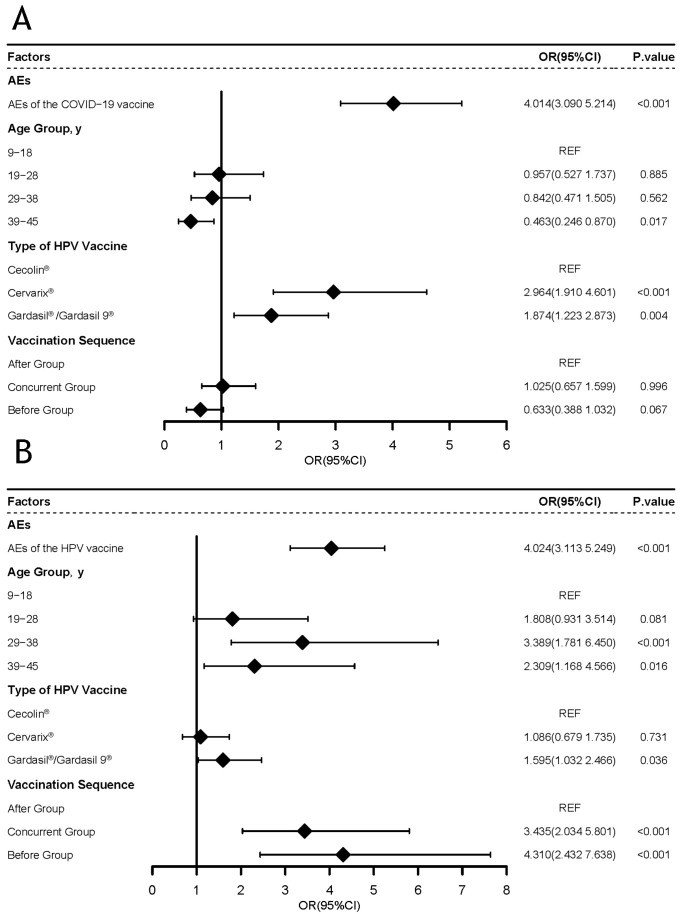
(**A**) Analysis of risk factors for the occurrence of AEs to HPV vaccines (n = 1416). (**B**) Analysis of risk factors for the occurrence of AEs to COVID-19 vaccines (n = 1416). The diamond symbol represents the OR (odds ratio) value.

To further analyze the factors influencing AEs in women after vaccination with the HPV vaccine, we performed a logistic regression analysis of the overall AEs of the HPV vaccine (Figure 2A). The variables we included in the regression were as follows: whether the COVID-19 vaccine AEs occurred, vaccination sequence, age, and HPV vaccine type. This study found that compared with women who did not experience overall AEs due to COVID-19 vaccines, women who experienced overall AEs caused by COVID-19 vaccines had a 4.014 times higher risk of HPV vaccine AEs (95% CI = 3.090–5.214, *p* < 0.001). The sequence of vaccination had no impact on the overall AEs of the HPV vaccine. This study also found that Cecolin^®^ corresponded to a lower risk of AEs {Cervarix^®^ (OR = 2.964, 95% CI = 1.910–4.601, *p* < 0.001); Gardasil^®^/Gardasil9^®^ (OR = 1.874, 95% CI = 1.223–2.873, *p* = 0.004)}.

Similarly, we analyzed the factors that influenced AEs among women after receiving the COVID-19 vaccine (Figure 2B). The results found that compared with women who did not experience overall AEs from the HPV vaccine, women who experienced overall AEs from the HPV vaccine had a 4.0424 times higher risk of COVID-19 vaccine AEs (95% CI = 3.113–5.249, *p* < 0.001). Further, the sequence of vaccination had an impact on the AEs from COVID-19 vaccines. Compared with the other two groups, the After group had a lower risk of COVID-19 vaccine AEs {Before group (OR = 4.310, 95% CI = 2.432–7.638, *p* < 0.001); Concurrent group (OR = 3.435, 95% CI = 2.034–5.801, *p* < 0.001)}. In terms of age, this study found that women aged 9–18 had a lower risk of COVID-19 vaccine AEs {29–38 years old (OR = 3.389, 95% CI = 1.781–6.450, *p* < 0.001); 39–45 years old (OR = 2.309, 95% CI = 1.168–4.566, *p* = 0.016)}.

## 4. Discussion

During the period of acute respiratory infectious disease, women may need to receive vaccines for HPV and other infectious diseases concurrently. However, most studies have evaluated two groups of people (those who received the HPV vaccine alone or the COVID-19 vaccine alone). Thus, there is less evidence on the safety of receiving both vaccines concurrently [25], and few relevant studies assess the Chinese population. To meet the above needs and increase opportunities for HPV vaccination, we designed a retrospective, descriptive study to observe the safety of concurrent immunization with the HPV vaccine and the COVID-19 vaccine and further determine the potential optimal vaccination sequence.

This study found that, except for menstrual irregularities, most AEs were short-term events lasting no more than seven days. The incidence of short-term AEs was lower after receiving the HPV vaccine than after receiving the COVID-19 vaccine. This result suggests that the HPV vaccine is safer than the COVID-19 vaccine. This result is consistent with the findings of Beniamino et al. [13], who found that the risk of serious adverse events (SAEs) was higher after vaccination with COVID-19 than after vaccination with the HPV vaccine. Compared to other studies involving concurrent immunization with the influenza vaccine and the COVID-19 vaccine, this study also observed a lower incidence of AEs after concurrent vaccination with the HPV vaccine and the COVID-19 vaccine (30.6% vs. 77%) [26]. Although this previous study was unrelated to the HPV vaccine, there is limited evidence on the safety of the HPV vaccine and the COVID-19 vaccine administered concurrently. To date, the limited evidence on this topic in the literature only explores rare AEs and lacks observations of comprehensive AEs [25]. Therefore, this previous research was used as reference evidence for the present study.

Additionally, compared with vaccination using the HPV vaccine alone, the incidence of AEs was lower when the HPV and COVID-19 vaccines were administered concurrently [27]. While the results of this study indicated a higher incidence of AEs from COVID-19 vaccination alone, compared to the results under concurrent administration [28], our study also considered menstrual irregularities in addition to analyzing common AEs. Without considering these symptoms, the results were similar to those from other clinical trials [29]. The above findings indicate that it is safe to receive the HPV vaccine and the COVID-19 vaccine concurrently.

Menstrual irregularities do not mean that the menstrual status is abnormal. This phenomenon may be caused by factors such as physiological or psychological status. Especially in adolescents, menstrual status may be closely related to pubertal development, these participants or their guardians cannot guarantee whether this phenomenon is menstrual abnormality [30]. However, we did find that girls aged 9–18 years old experienced menstrual irregularities after receiving the HPV or COVID-19 vaccines. Therefore, we used the 9- to 18-year-old group and the 29- to 38-year-old group as reference categories to analyze the relationship between age and the incidence of AEs (Figure 2 and Figure S1). We found that for COVID-19 vaccines, 29–38 years old is a risk factor for AEs. In future studies, it may be beneficial to increase the sample size of minors to further investigate this aspect.

To determine the optimal vaccination sequence, this study divided the population into three groups: (1) Those administered the HPV vaccine before the COVID-19 vaccine (Before group), (2) those vaccinated concurrently with both vaccines (Concurrent group), and (3) those administered the HPV vaccine after the COVID-19 vaccine (After group). We found that the best vaccination sequence was the After group. For HPV vaccines, the sequence of vaccination had no impact on overall AEs, but the incidence of individual AEs was different. This result was clearly reflected in the lower incidence of pain at the vaccination site among those in the After group. However, the reason for this significant difference remains unclear. We speculate that this difference may be due to the higher severity of AEs after receiving the COVID-19 vaccine than after receiving the HPV vaccine. Under this background, changes in people’s subjective perceptions of AEs could lower the sensitivity threshold for reporting AEs from HPV vaccines. The severity of AEs could be analyzed in future studies.

The present research found the After group to be safer. However, some studies have found that the prevalence of HPV is highest among women under 34 years old [31], thus, receiving the HPV vaccine as soon as possible may effectively prevent cervical cancer. Moreover, the AEs of concurrent vaccination with the HPV vaccine and the COVID-19 vaccine are mostly short-term reactions. Therefore, combined with the above factors, we still recommend receiving the HPV vaccine as early as possible to reduce the risk of HPV infection.

In addition, the present study found that the Cecolin^®^ vaccine was associated with a lower risk of AEs (Figure 2A) (Table S6). This result is consistent with the outcomes of previous clinical trials [27] and highlights the safety of the Cecolin^®^ vaccine. Due to its cost-effectiveness, including this vaccine in their national immunization plans would also benefit low—and middle-income countries.

During the pre-investigated, we found that the follow-up time for respondents was approximately 5–8 min. If the follow-up time was too long, respondents were unwilling to continue answering. Additionally, when asked, “Have you experienced any adverse events 30 days after vaccination?” some respondents answered, “I don’t remember” and refused to continue. We suspect this is because people focus more on “30 days after vaccination” rather than “adverse events”. Therefore, we removed “30 days”. We found that asking, “Have you experienced any adverse events after vaccination?” received more responses. This increased response rate may also lead to suggestive questioning.

There are several limitations in our study. First, this study did not include women who received the HPV vaccine alone. Instead, we used women who received both the HPV vaccine and the COVID-19 vaccine as their own controls. However, due to China’s large-scale COVID-19 vaccination program, data on those who received the HPV vaccine alone remains challenging. Considering the availability of data, we compared the results with those of other studies on HPV vaccination alone and found that our results were consistent. Additionally, it may be possible further to select external populations as controls in the future. Second, COVID-19 vaccines allow mixed vaccination [32]. Thus, we could not divide the population according to the type of COVID-19 vaccine. Consequently, COVID-19 vaccine types were not included when analyzing the influencing factors of the COVID-19 vaccine AEs. Finally, this study used only telephone conversations/interviews to collect information. Due to the high incidence of phone fraud and low level of trust among phone survey participants at present, the data we collected were subjective and self-reported by participants. Moreover, this is a retrospective study. Most participants could only answer if the AEs occurred after the HPV or COVID-19 vaccine. Thus, we cannot guarantee the exact timing of possible AEs. There was also a lack of objective indicators for clinical testing. However, other studies using this approach have investigated similar events [33,34]. Face-to-face surveys could be used for regular follow-ups in the future.

Despite these limitations, we believe the present findings represent an essential addition to the safety profile of concurrent vaccination with HPV and respiratory infectious disease vaccines. Our research results will help promote the inclusion of HPV vaccines in the national immunization programs of low- and middle-income countries, especially during outbreaks of acute respiratory infectious diseases. These results could also be used as supporting evidence to improve the global coverage of HPV vaccines.

## 5. Conclusions

Our research found that concurrent vaccination with the HPV vaccine and the COVID-19 vaccine is safe. However, we observed a lower risk of AEs from HPV vaccination after COVID-19 vaccination. The specific symptom was pain at the vaccination site, but medical professionals should also consider the impacts of vaccination on the female endocrine system, such as menstrual irregularities. Considering the effectiveness of the HPV vaccine, we recommend that women receive the HPV vaccine and respiratory infectious disease vaccine as soon as possible during outbreaks of respiratory infectious diseases. At the same time, as the Cecolin^®^ vaccine is affordable and of high quality, low- and middle-income countries should include this vaccine in their national immunization plans for young women.

## Figures and Tables

**Table 1:** Description of baseline information.

	Total n = 1416	Before Group ^1^ n = 773	Concurrent Group ^2^ n = 418	After Group ^3^ n = 225	*p*
Age (in years)					<0.001
Median (IQR) ^4^	33 (27–38)	33 (25–37)	32 (27–38)	35 (30–40)	
Age Group, y					<0.001
9–18	68 (4.8)	43 (5.6)	25 (6.0)	0 (0.0)	
19–28	349 (24.7)	204 (26.4)	98 (23.4)	47 (20.9)	
29–38	675 (47.7)	369 (47.7)	200 (47.9)	106 (47.1)	
39–45	324 (22.9)	157 (20.3)	95 (22.7)	72 (32.0)	
Type of HPV vaccine					<0.001
Cecolin^®^	373 (26.3)	0 (0.0)	194 (46.4)	179 (79.6)	
Cervarix^®^	281 (19.8)	193 (25.0)	47 (11.2)	41 (18.2)	
Gardasil^®^/Gardasil 9^®^	762 (53.8)	580 (75.0)	177 (42.3)	5 (2.2)	
Adverse events (AEs)					<0.001
Occurred with both	213 (15.0)	130 (16.8)	71 (17.0)	12 (5.3)	
Occurred only witrred only with COVID-19	231 (16.3)	164 (21.2)	57 (13.6)	10 (4.4)	
Occurred with neither	792 (55.9)	384 (49.7)	237 (56.7)	171 (76.0)	

^1^ Received the HPV vaccine before COVID-19 vaccination. ^2^ The HPV vaccine and COVID-19 vaccines were administered concurrently. ^3^ Received the HPV vaccine after COVID-19 vaccination. ^4^ Rank sum test.

**Table 2:** AEs following HPV vaccination and COVID-19 vaccination (n = 1416) (%).

	Occurred with Both ^1^	Occurred only with HPV ^2^	Occurred only with COVID-19 ^3^	*p*
Total AEs	213 (15.0)	180 (12.7)	231 (16.3)	0.014
Local AEs	123 (8.7)	148 (10.5)	166 (11.7)	0.337
Pain	119 (8.4)	139 (9.8)	151 (10.7)	0.518
Induration	0 (0.0)	4 (0.3)	3 (0.2)	1.000
Redness	2 (0.1)	6 (0.4)	4 (0.3)	0.754
Swelling	11 (0.8)	34 (2.4)	30 (2.1)	0.708
Rash	0 (0.0)	1 (0.1)	0 (0.0)	1.000
Pruritus	0 (0.0)	0 (0.0)	6 (0.4)	0.031
Systemic AEs	49 (3.5)	95 (6.7)	150 (10.6)	<0.001
Fever	1 (0.1)	2 (0.1)	17 (1.2)	<0.001
Diarrhea	0 (0.0)	0 (0.0)	6 (0.4)	0.031
Vomiting	0 (0.0)	1 (0.1)	1 (0.1)	1.000
Nausea	0 (0.0)	1 (0.1)	4 (0.3)	0.375
Muscle pain	0 (0.0)	0 (0.0)	7 (0.5)	0.016
Headache	0 (0.0)	0 (0.0)	3 (0.2)	0.250
Syncope	0 (0.0)	0 (0.0)	0 (0.0)	1.000
Cough	0 (0.0)	2 (0.1)	1 (0.1)	1.000
Itching ^4^	0 (0.0)	2 (0.1)	0 (0.0)	0.500
Insomnia	0 (0.0)	0 (0.0)	7 (0.5)	0.016
Drowsiness	1 (0.1)	8 (0.6)	38 (2.7)	<0.001
Anaphylaxis	1 (0.1)	2 (0.1)	10 (0.7)	0.039
Fatigue	5 (0.4)	4 (0.3)	54 (3.8)	<0.001
Pain ^5^	0 (0.0)	1 (0.1)	8 (0.6)	0.039
Menstrual irregularities	16 (1.1)	105 (7.4)	43 (3.0)	<0.001
Short cycle	0 (0.0)	0 (0.0)	1 (0.1)	1.000
Long cycle	0 (0.0)	2 (0.1)	1 (0.1)	1.000
Irregular menstrual cycles	5 (0.4)	26 (1.8)	18 (1.3)	0.291
Prolonged menstruation duration	0 (0.0)	8 (0.6)	1 (0.1)	0.039
Light menstrual bleeding	4 (0.2)	23 (1.6)	12 (0.8)	0.091
Heavy menstrual bleeding (HMB)	0 (0.0)	6 (0.4)	0 (0.0)	0.091
Dysmenorrhea	0 (0.0)	3 (0.2)	3 (0.2)	1.000
Intermenstrual bleeding (IMB)	1 (0.1)	1 (0.1)	0 (0.0)	1.000

^1^ AEs that occurred after receiving both vaccines. ^2^ AEs that occurred only with the HPV vaccine but not with the COVID-19 vaccine. ^3^ AEs that occurred only with the COVID-19 vaccine but not with the HPV vaccine. ^4^ Non-vaccination site itching. ^5^ Pain in non-vaccination areas other than muscle pain, joint pain, or headache.

**Table 3:** Comparison of AEs after HPV vaccination with different sequences of vaccination (n = 1416) (%).

	Before Group ^1^ n = 773	Concurrent Group ^2^ n = 418	After Group ^3^ n = 225	*p*
Total AEs	225 (29.1)	124 (29.7)	44 (19.6)	0.011
9–18 (n = 68)	14 (32.6)	7 (28.0)	0 (0.0)	0.789
19–28 (n = 349)	69 (33.8)	27 (27.6)	9 (19.1)	0.116
29–38 (n = 675)	110 (29.8)	70 (35.0)	29 (27.4)	0.301
39–45 (n = 324)	32 (20.4)	20 (21.1)	6 (8.3)	0.057
Local AEs	172 (22.3)	81 (19.4)	18 (8.0)	<0.001
Pain	163 (21.1)	79 (18.9)	16 (7.1)	<0.001
Induration	2 (0.3)	2 (0.5)	0 (0.0)	0.640
Redness	3 (0.4)	3 (0.7)	2 (0.9)	0.437
Swelling	31 (4.0)	8 (1.9)	6 (2.7)	0.126
Rash	0 (0.0)	1 (0.2)	0 (0.0)	0.454
Pruritus	0 (0.0)	0 (0.0)	0 (0.0)	1.000
Systemic AEs	69 (8.9)	50 (12.0)	25 (11.1)	0.217
Fever	2 (0.3)	1 (0.2)	0 (0.0)	1.000
Diarrhea	0 (0.0)	0 (0.0)	0 (0.0)	1.000
Vomiting	0 (0.0)	1 (0.2)	0 (0.0)	0.454
Nausea	0 (0.0)	1 (0.2)	0 (0.0)	0.454
Muscle pain	0 (0.0)	0 (0.0)	0 (0.0)	1.000
Headache	0 (0.0)	0 (0.0)	0 (0.0)	1.000
Syncope	0 (0.0)	0 (0.0)	0 (0.0)	1.000
Cough	1 (0.1)	1 (0.2)	0 (0.0)	1.000
Itching ^4^	0 (0.0)	2 (0.5)	0 (0.0)	0.112
Insomnia	0 (0.0)	0 (0.0)	0 (0.0)	1.000
Drowsiness	8 (1.0)	1 (0.2)	0 (0.0)	0.148
Acute anaphylaxis	0 (0.0)	1 (0.2)	2 (0.9)	0.026
Fatigue	8 (1.0)	1 (0.2)	0 (0.0)	0.148
Pain ^5^	0 (0.0)	1 (0.2)	0 (0.0)	0.454
Menstrual irregularities	54 (7.0)	44 (10.5)	23 (10.2)	0.069
Short cycle	0 (0.0)	0 (0.0)	0 (0.0)	1.000
Long cycle	2 (0.3)	0 (0.0)	0 (0.0)	0.677
Irregular menstrual cycles	17 (2.2)	9 (2.2)	5 (2.2)	1.000
Prolonged menstruation duration	0 (0.0)	4 (1.0)	4 (1.8)	0.001
Light menstrual bleeding	12 (1.6)	11 (2.6)	4 (1.8)	0.443
HMB	1 (0.1)	3 (0.7)	1 (0.9)	0.108
Dysmenorrhea	0 (0.0)	1 (0.2)	2 (0.9)	0.026
IMB	1 (0.1)	1 (0.2)	0 (0.0)	1.000

^1^ Received the HPV vaccine before COVID-19 vaccination. ^2^ The HPV and COVID-19 vaccines were administered concurrently. ^3^ Received the HPV vaccine after COVID-19 vaccination. ^4^ Non-vaccination site itching. ^5^ Pain in non-vaccination areas other than muscle pain, joint pain, or headache.

**Table 4:** Comparison of AEs after COVID-19 vaccination with different sequences of vaccination (n = 1416) (%).

	Before Group ^1^ n = 773	Concurrent Group ^2^ n = 418	After Group ^3^ n = 225	*p*
Total AEs	294 (38)	128 (30.6)	22 (9.8)	<0.001
9–18 (n = 68)	9 (20.9)	5 (20.0)	0 (0)	1.000
19–28 (n = 349)	71 (34.8)	23 (23.5)	5 (10.6)	0.002
29–38 (n = 675)	162 (43.9)	78 (39.0)	12 (11.3)	<0.001
39–45 (n = 324)	52 (33.1)	22 (23.2)	5 (6.9)	<0.001
Local AEs	202 (26.1)	74 (17.7)	13 (5.8)	<0.001
Pain	188 (24.6)	69 (16.5)	13 (5.8)	<0.001
Induration	3 (0.4)	0 (0.0)	0 (0.0)	0.736
Redness	3 (0.4)	2 (0.5)	1 (0.4)	1.000
Swelling	28 (3.6)	12 (2.9)	1 (0.4)	0.041
Rash	0 (0.0)	0 (0.0)	0 (0.0)	1.000
Pruritus	6 (0.8)	0 (0.0)	0 (0.0)	0.135
Systemic AEs	120 (15.5)	67 (16.0)	12 (5.3)	<0.001
Fever	7 (0.9)	9 (2.2)	2 (0.9)	0.172
Diarrhea	2 (0.3)	4 (1.0)	0 (0.0)	0.202
Vomiting	1 (0.1)	0 (0.0)	0 (0.0)	1.000
Nausea	1 (0.1)	3 (0.7)	0 (0.0)	0.201
Muscle pain	4 (0.5)	2 (0.5)	1 (0.4)	1.000
Headache	2 (0.3)	1 (0.2)	0 (0.0)	1.000
Syncope	0 (0.0)	0 (0.0)	0 (0.0)	1.000
Cough	0 (0.0)	0 (0.0)	1 (0.4)	0.159
Itching ^4^	0 (0.0)	0 (0.0)	0 (0.0)	1.000
Insomnia	4 (0.5)	3 (0.7)	0 (0.0)	0.592
Drowsiness	26 (3.4)	12 (2.9)	1 (0.4)	0.062
Acute anaphylaxis	7 (0.9)	3 (0.7)	1 (0.4)	0.920
Fatigue	36 (4.7)	20 (4.8)	3 (1.3)	0.066
Pain ^5^	5 (0.6)	3 (0.7)	0 (0.0)	0.702
Menstrual irregularities	38 (4.9)	19 (10.5)	2 (0.9)	0.026
Short cycle	0 (0.0)	1 (0.2)	0 (0.0)	0.454
Long cycle	1 (0.1)	0 (0.0)	0 (0.0)	1.000
Irregular menstrual cycles	16 (2.1)	5 (1.2)	2 (0.9)	0.356
Prolonged menstruation duration	0 (0.0)	1 (0.2)	0 (0.0)	0.454
Light menstrual bleeding	11 (1.4)	5 (1.2)	0 (0.0)	0.193
HMB	0 (0.0)	0 (0.0)	0 (0.0)	1.000
Dysmenorrhea	2 (0.3)	12 (0.)	0 (0.0)	1.000
IMB	1 (0.1)	0 (0.0)	0 (0.0)	1.000

^1^ Received the HPV vaccine before COVID-19 vaccination. ^2^ The HPV and COVID-19 vaccines were administered concurrently. ^3^ Obtained the HPV vaccine after COVID-19 vaccination. ^4^ Itching at non-vaccination sites. ^5^ Pain in non-vaccination areas other than muscle pain, joint pain, or headache.

## Data Availability

The data presented in this study are available on request from the corresponding author.

## Supplementary Materials

The following supporting information can be downloaded at: https://www.mdpi.com/article/10.3390/vaccines12060673/s1, Table S1. Survey instrument for adverse events (AEs) with the HPV vaccine and the COVID-19 vaccine (relevant parts of this study). Table S2. Descriptive baseline information of the study participants (n = 2682). Table S3. Definitions of outcomes. Table S4. Comparison of AEs to the HPV vaccine with different sequences of vaccination (n = 1416) (%). Table S5. Comparison of AEs to the COVID-19 vaccine with varying sequences of vaccination (n = 1416) (%). Table S6: AEs following different types of HPV vaccination (n = 1416) (%). Figure S1. (A) Analysis of risk factors for the occurrence of AEs to HPV vaccines (n = 1416). (B) Analysis of risk factors for the occurrence of AEs to COVID-19 vaccines (n = 1416).
