# Supplementary material for: Safety Assessment of Concurrent Vaccination with the HPV Vaccine and the COVID-19 Vaccine in Fujian Province, China: A Retrospective Study
Source: Vaccines (Basel). 2024 Jun 18;12(6):673. doi: 10.3390/vaccines12060673 (PMC11209349; doi:10.3390/vaccines12060673)
Supplement: Supplementary file 1 [file vaccines-12-00673-s001.zip › vaccines-2978803-supplement.pdf]

Table S1. Survey instrument for adverse events (AEs) of with the human papillomavirus (HPV) vaccine and the coronavirus disease 2019 (COVID-19) vaccine (relevant parts of the study).

Survey instrument
Hello, are you Ms. XXX? We are staff members from Fujian Maternal and Child Health Hospital. We would like to follow up with you on your physical health after receiving the HPV vaccine and COVID-19 vaccine for vaccine safety research. This research was approved by the Ethics Committee of Fujian Maternal and Child Health Hospital (2023KYLLR01025). We will de-identify your data and ensure that your personal privacy will not be disclosed. The data will use for statistical analysis only. Would you like to take part in our research and answer a few questions?<br>☐Yes<br>☐No
The researchers checked for personally information such as the participant identity card [ID], name, date of birth (age), address, vaccine name, vaccination manufacturer, vaccination date and previous history of HPV infection to confirm the identity of the participants.
Questions about HPV vaccine
1. Have you completed all HPV vaccination procedures?<br>☐Yes,_____(If so, the researchers need to check the vaccination time.)<br>☐No
2. Did you experience any local AEs after receiving the HPV vaccine, such as pain, induration, redness, swelling, rash and pruritus at the injection site?<br>☐Yes,_____(If so, what are the AEs? When the AEs occurred? How long did it last? What is the severity level?)<br>☐No
3. Did you experience any systemic AEs after receiving the HPV vaccine, such as fever, diarrhea, vomiting, nausea, muscle pain, headache, syncope, cough, itching, insomnia, drowsiness, anaphylaxis, fatigue and pain?<br>☐Yes,_____(If so, what are the AEs? When the AEs occurred? How long did it last? What is the severity level?)<br>☐No
4. Did you experience menstrual irregularities after receiving the HPV vaccine, such as short cycle, long cycle, irregular menstrual cycles, prolonged menstruation duration, light menstrual bleeding, heavy menstrual bleeding (HMB), dysmenorrhea, intermenstrual bleeding (IMB)?<br>☐Yes,_____(If so, what are the AEs? When the AEs occurred? How long did it last? What is the severity level?)<br>☐No
Questions about COVID-19 vaccine
1. Have you completed all COVID-19 vaccination procedures?<br>☐Yes,_____(If so, the researchers need to check the vaccination time)<br>☐No
2. Did you experience any local AEs after receiving the COVID-19 vaccine, such as pain, induration, redness, swelling, rash and pruritus at the injection site?<br>☐Yes,_____(If so, what are the AEs? When the AEs occurred? How long did it last? What is the severity level?)<br>☐No
3. Did you experience any systemic AEs after receiving the COVID-19 vaccine, such as fever, diarrhea, vomiting, nausea, muscle pain, headache, syncope, cough, itching, insomnia, drowsiness, anaphylaxis, fatigue and pain?<br>☐Yes,_____(If so, what are the AEs? When the AEs occurred? How long did it last? What is the severity level?)<br>☐No
4. Did you experience menstrual irregularities after receiving the COVID-19 vaccine, such as short cycle, long cycle, irregular menstrual cycles, prolonged menstruation duration, light menstrual bleeding, HMB, dysmenorrhea, IMB?<br>☐Yes,_____(If so, what are the AEs? When the AEs occurred? How long did it last? What is the severity level?)<br>☐No
The follow-up is over, thank you for your patience and cooperation.

Table S2. Descriptive baseline information of the study participants (n = 2682).

	Participants (%)
Total (n)	2682 (100)
Age (in years)	
Median (IQR)	33 (26-38)
Residence[1]	
Fuzhou city Districts	2349 (87.60)
Fuzhou Counties	150 (5.60)
Not Fuzhou (within Fujian Province)	83 (3.10)
Unknown	100 (3.70)
Type of HPV vaccine	
Cecolin®	570 (21.30)
Cervarix®	527 (19.60)
Gardasil®/Gardasil 9®	1585 (59.10)
Previous history of HPV infection	
Negative	423 (15.80)
Positive	67 (2.50)
Unknown	2192 (81.70)

[1] Fuzhou is divided into five city districts (Gulou, Jinan, Cangshan, Taijiang, Mawei) and eight counties (Changle, Fuqing, Minhou, Luoyuan, Lianjiang, Pingtan, Yongtai, Minqing).

Table S3. Definitions of outcomes.

Outcome	Definition
Adverse events (AEs)[1]	Adverse events refer to all adverse medical events that occur in participants after vaccination, which may manifest as symptoms, signs, or illnesses, but may not necessarily be related to the vaccine
Local AEs[1]	Local adverse events refer to adverse events occurring at the vaccination site, such as pain, induration, redness, swelling, rash, and itching.
Systemic AEs[1]	Systemic reactions included fever, diarrhea, vomit, nausea, myalgia, headache, syncope, cough, itching, insomnia, drowsiness, allergic reaction, fatigue, pain, other.
Menstrual irregularities[2]	It is defined as bleeding from the uterine corpus that is irregularities in duration, volume, frequency, and/or regularity, and has been present for the majority of the preceding 6 months.
Menstrual cycle	Menstrual cycle is defined as the number of days between the first day of the previous menstrual cycle and the first day of the current menstrual cycle.<br>Short cycle (Frequent) is defined as <24 days.<br>Long cycle (Infrequent) is defined as >38 days.
Regularity	Regular in a way that you could usually predict about when the next period would start.<br>Irregular menstrual cycles are defined as ≥8-10 day variations, depending on age (18-25 years ≥10 days; 26-41 years ≥8 days; 42-45 years ≥10 days).
Menstrual duration	Menstrual duration is defined as the number of days a person bleeds during their menstrual period.<br>Prolonged menstrual duration is defined as menstrual duration > 8 days.
Flow volume	Flow volume is defined as the amount of bleeding per menstrual period, which is determined subjectively by each individual.<br>Flow volume is divided into light menstrual bleeding and heavy menstrual bleeding (HMB).<br>HMB is defined as excessive menstrual blood loss, which interferes with a woman's physical, social, emotional and/or material quality of life.
Dysmenorrhea	Dysmenorrhea is defined as abdominal pain during menstruation.
Intermenstrual Bleeding (IMB)	Spontaneous bleeding occurring between menstrual periods.

[1] According to Guidelines for grading standards of adverse events in clinical studies of prophylactic vaccines (No. 102, 2019) issued by the China National Medical Products of Administration. [2] According to the guidelines updated by the

International Federation of Obstetrics and Gynecology (FIGO) in 2018.

Table S4. Comparison of AEs to the HPV vaccine within different sequences of vaccination (n = 1416) (%).

	**Before group[1] n=773**	**Concurrent group[2] n=418**	**After group[3] n=225**	**P**	**$P_{1,2}$**	**$P_{1,3}$**	**$P_{2,3}$**
Total AEs	225 (29.1)	124 (29.7)	44 (19.6)	0.011	0.842	0.005	0.006
Local AEs	172 (22.3)	81(19.4)	18 (8.0)	<0.001	0.266	<0.001	<0.001
Pain	163 (21.1)	79 (18.9)	16 (7.1)	<0.001	0.407	<0.001	<0.001
Systemic AEs	69 (8.9)	50 (12.0)	25 (11.1)	0.217			
Acute anaphylaxis	0 (0.0)	1 (0.2)	2 (0.9)	0.026	0.351	0.051	0.281
Menstrual irregularities	54 (7.0)	44 (10.5)	23 (10.2)	0.069			
Prolonged menstruation duration	0 (0.0)	4 (1.0)	4 (1.8)	0.001	0.015	0.003	0.460
Dysmenorrhea	0 (0.0)	1 (0.2)	2 (0.9)	0.026	0.351	0.051	0.281

[1] Received the HPV vaccine before COVID-19 vaccination. [2] The HPV vaccine and COVID-19 vaccines were administered concurrently. [3] Received the HPV vaccine after COVID-19 vaccination.

Table S5. Comparison of AEs to the COVID-19 vaccine in with different sequences of vaccination (n = 1416) (%).

	**Before group[1] n =773**	**Concurrent group[2] n=418**	**After group[3] n=225**	**P**	**$P_{1,2}$**	**$P_{1,3}$**	**$P_{2,3}$**
Total AEs	294 (38)	128 (30.6)	22 (9.8)	<0.001	0.011	<0.001	<0.001
19-28 (n=349)	71 (34.8)	23 (23.5)	5 (10.6)	0.002	0.048	<0.001	0.075
29-38 (n=675)	162 (43.9)	78 (39.0)	12 (11.3)	<0.001	0.286	<0.001	<0.001
39-45 (n=324)	52 (33.1)	22 (23.2)	5 (6.9)	<0.001	0.118	<0.001	0.005
Local AEs	202 (26.1)	74 (17.7)	13 (5.8)	<0.001	0.001	<0.001	<0.001
Pain	24.6 (188)	69 (16.5)	13 (5.8)	<0.001	0.002	<0.001	<0.001
Swelling	3.6 (28)	12 (2.9)	1 (0.4)	0.041	0.509	0.011	0.040
Systemic AEs	15.5 (120)	67 (16.0)	12 (5.3)	<0.001	0.867	<0.001	<0.001
Menstrual irregularities	4.9 (38)	19 (10.5)	2 (0.9)	0.026	0.780	0.006	0.017

[1] Received the HPV vaccine before COVID-19 vaccination. [2] The HPV vaccine and COVID-19 vaccines were administered concurrently. [3] Received the HPV vaccine after COVID-19 vaccination.

Table S6. AEs following different types of HPV vaccination (n = 1416) (%).

	**Cecolin®[1] n=373**	**Cervarix®[2] n=281**	**Gardasil®/ Gardasil 9®[3] n=762**	**P**	**$P_{1,2}$**	**$P_{1,3}$**	**$P_{2,3}$**
Total AEs	67 (18.0)	98 (34.9)	228 (29.9)	<0.001	<0.001	<0.001	0.132
9-18 (n=68)	0 (0.0)	5 (45.5)	16 (28.6)	0.517			
19-28 (n=349)	6 (9.7)	6 (26.1)	93 (35.2)	<0.001	0.078	<0.001	0.494
29-38 (n=675)	43 (22.1)	69 (44.5)	97 (29.8)	<0.001	<0.001	0.066	0.002
39-45 (n=324)	18 (15.7)	18 (19.6)	22 (18.8)	0.758			
Local AEs	37 (9.9)	80 (28.5)	154 (20.2)	＜0.001	＜0.001	<0.001	0.006
Pain	34 (9.1)	77 (27.4)	147 (19.3)	＜0.001	＜0.001	<0.001	0.005
Induration	0 (0.0)	1 (0.4)	3 (0.4)	0.653			
Redness	1 (0.3)	5 (1.6)	2 (0.3)	0.016	0.089	1.000	0.018
Swelling	7 (1.9)	15 (5.3)	23 (3.0)	0.038	0.017	0.326	0.093
Rash	1 (0.3)	0 (0.0)	0 (0.0)	0.462			
Pruritus	0 (0.0)	0 (0.0)	0 (0762)	1.000			
Systemic AEs	31 (8.3)	24 (8.5)	89 (11.7)	0.126			
Fever	0 (0.0)	1 (0.0)	3 (0.4)	0.429			
Diarrhea	0 (0.0)	0 (0.0)	0 (0.0)	1.000			
Vomiting	0 (0.0)	0 (0.0)	1 (0.1)	1.000			
Nausea	0 (0.0)	0 (0.0)	1 (0.1)	1.000			
Muscle pain	0 (0.0)	0 (0.0)	0 (0.0)	1.000			
Headache	0 (0.0)	0 (0.0)	0 (0.0)	1.000			
Syncope	0 (0.0)	0 (0.0)	0 (0.0)	1.000			
Cough	1 (0.3)	1 (0.4)	0 (0.0)	0.213			
Itching[4]	0 (0.0)	2 (0.7)	0 (0.0)	0.039	0.184	1.000	0.072
Insomnia	0 (0.0)	0 (0.0)	0 (0.0)	1.000			
Drowsiness	0 (0.0)	1 (0.4)	8 (1.2)	0.083			
Acute anaphylaxis	3 (0.8)	0 (0.0)	0 (0.0)	0.026	0.264	0.035	1.000
Fatigue	1 (0.3)	1 (0.4)	7 (0.9)	0.3491			
Pain[5]	1 (0.4)	0 (0.0)	0 (0.0)	0.462			
Menstrual irregularities	26 (7.0)	20 (7.1)	75 (9.8)	0.174			
Short cycle (Frequent)	0 (0.0)	0 (0.0)	0 (0.0)	1.000			
Long cycle (Infrequent)	0 (0.0)	0 (0.0)	2 (0.3)	1.000			
Irregular menstrual cycles	5 (1.3)	2 (0.7)	24 (3.1)	0.022	0.705	0.074	0.024
Prolonged menstruation duration	6 (1.6)	1 (0.4)	1 (0.1)	0.007	0.248	0.006	0.466
Light menstrual bleeding	10 (2.7)	3 (1.1)	14 (1.8)	0.307			
HMB	3 (0.8)	0 (0.0)	3 (0.4)	0.349			
Dysmenorrhea	2(0.5)	0 (0.0)	1 (0.1)	0.274			
IMB	0 (0.0)	1 (0.4)	1 (0.1)	0.427			

[4] Itching: non-vaccination site itching. [5] Pain: pain in non-vaccination areas other than muscle pain, joint pain, and headache.

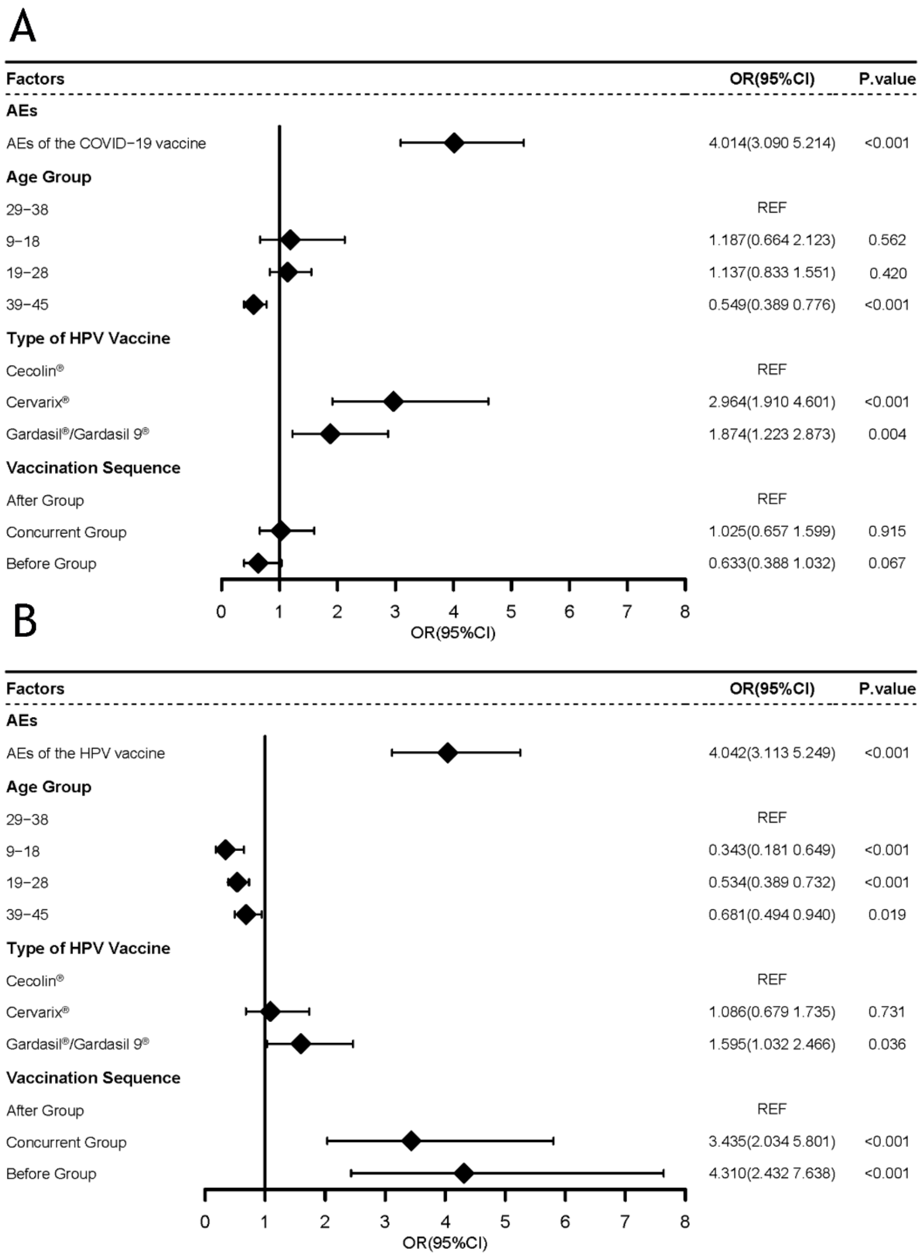

Figure S1. (**A**) Analysis of risk factors for the occurrence of AEs to HPV vaccines (n = 1416). (**B**) Analysis of risk factors for the occurrence of AEs to COVID-19 vaccines (n = 1416).
